# Should prevention of falls start earlier? Co-ordinated analyses of harmonised data on falls in middle-aged adults across four population-based cohort studies

**DOI:** 10.1371/journal.pone.0201989

**Published:** 2018-08-07

**Authors:** Geeske Peeters, Natasja M. van Schoor, Rachel Cooper, Leigh Tooth, Rose Anne Kenny

**Affiliations:** 1 Global Brain Health Institute, University of California San Francisco, California, United States of America | Trinity College Dublin, Dublin, Ireland; 2 Faculty of Medicine, School of Public Health, The University of Queensland, Brisbane, Queensland, Australia; 3 Amsterdam Public Health Research institute, Department of Epidemiology and Biostatistics, VU University Medical Center, Amsterdam, The Netherlands; 4 MRC Unit for Lifelong Health and Ageing at UCL, London, United Kingdom; 5 Mercer’s Institute for Successful Ageing, St James’s Hospital, Dublin, Ireland; 6 The Irish Longitudinal Study on Ageing, Trinity College, Dublin, Ireland; University of Florence, ITALY

## Abstract

The prevalence of risk factors for falls increases during middle-age, but the prevalence of falls in this age-range is often overlooked and understudied. The aim was to calculate the prevalence of falls in middle-aged adults (aged 40–64 years) from four countries.

Data were from four population-based cohort studies from Australia (Australian Longitudinal Study on Women’s Health, n = 10556, 100% women, 51–58 years in 2004), Ireland (The Irish Longitudinal Study on Ageing, n = 4968, 57.5% women, 40–64 years in 2010), the Netherlands (Longitudinal Aging Study Amsterdam, n = 862, 51.6% women, 55–64 years in 2012–13) and Great Britain (MRC National Survey of Health and Development, n = 2821, 50.9% women, 53 years in 1999). In each study, falls assessment was based on recall of any falls in the past year. The prevalence of falls was calculated for the total group, for each country, for men and women separately, and for 5-year age-bands. The prevalence was higher in Australia (27.8%, women only) and the Netherlands (25.1%) than in Ireland (17.6%) and Great Britain (17.8%, p<0.001). Women (27.0%) had higher prevalences than men (15.2%, p<0.001). The prevalence increased from 8.7% in 40–44 year olds to 29.9% in 60–64 year olds in women, and from 14.7% in 45–49 year olds to 15.7% in 60–64 year olds in men. Even within 5-year age-bands, there was substantial variation in prevalence between the four cohorts. Weighting for age, sex and education changed the prevalence estimates by less than 2 percentage points.

The sharp increase in prevalence of falls in middle-age, particularly among women supports the notion that falls are not just a problem of old age, and that middle-age may be a critical life stage for preventive interventions.

## Introduction

The high prevalence and burden of falls in older adults has been widely described [1, 2]. Meta-analyses suggest that interventions targeting adults aged 65+ could lower the risk of falls by up to 30% [3]. But despite this, population injury rates for falls and injuries from falls continue to rise independent of the ageing of the population [4, 5], suggesting a failure of intervention strategies and/or a change in risk factor profiles of those who fall. The rising rates in injuries from falls and the ageing of the population has led to calls for new approaches to falls prevention [6]. Current falls prevention guidelines predominantly focus on adults over the age of 65 with a high falls risk based on presence of risk factors [7, 8]. While this approach is sensible from the point of view of providing care to those with the highest need, it ignores the opportunity for early preventive interventions.

A past fall is the strongest predictor of a future fall [9], suggesting that primary prevention is important. The second strongest predictor of falls is abnormality of gait or balance [9]. At the population-level, onset of declines in balance ability and other measures of physical functioning are typically observed between ages 40 and 60 [10, 11]. The prevalence of other risk factors for falls, such as syncope, dizziness and chronic conditions, also increase after the age of 50 [12, 13], particularly in women after menopause. These findings suggest that middle-age may be a critical life stage for early interventions for falls prevention.

A first step in exploring opportunities for preventive strategies at younger ages is to establish the prevalence of falls in middle-aged adults. To date, few population-based studies have examined the prevalence of falls in this age group. However, those studies that have reported on this, report prevalences ranging from 11 to 30% [4, 14]. For example, in the 2008 U.S. National Health Interview Survey, the prevalence of falls in the past year among adults aged 45–64 years was 11.4% [4]. In the middle-aged cohort of the Australian Longitudinal Study on Women’s Health, the prevalence of falls varied between 21 and 31% between ages 53–58 and 62–67 [15]. Studies in older adults suggest that country differences exist in the prevalence of falls [2, 16–18], indicating that published prevalences cannot be automatically extrapolated to other countries. Moreover, these studies do not show how the prevalence changes during middle-age.

The aim of this study is to calculate the prevalence of falls in middle-aged adults (aged 40–64 years) from four countries, and to examine how the prevalence changes during middle-age. Data were used from population-based cohort studies from Australia, the Netherlands, Great Britain and Ireland. These studies were selected based on the availability of falls data measured using similar methods in the relevant age-range. Previous publications from these cohort studies reported on the prevalence of falls in older adults only (e.g. [19, 20]) or reported the prevalence for the 50–64 year old group as a whole [21].

## Materials and methods

### Study sample

The Australian Longitudinal Study on Women’s Health (ALSWH) is a prospective study of the health and well-being of four generations of women [22, 23]. Samples were randomly drawn from the national Medicare health insurance database, which includes all Australian citizens and permanent residents, with intentional over-representation of women from rural and remote areas [22]. The study was approved by Ethics Committees of the Universities of Newcastle and Queensland. All participants provided informed consent. In 1996, 13714 participants from the mid-age cohort (born 1946–51) returned the baseline survey (response rate 54%). Follow-up surveys have been completed at approximately 3-year intervals. Falls data were available from the 2004 survey onward. For the current analysis, data were used from 10556 women aged 51–58 years in 2004, 9547 women aged 53–61 years in 2007 and 8995 women aged 57–64 years in 2010 with complete data on age, education and falls.

The Longitudinal Ageing Study Amsterdam (LASA) is an ongoing interdisciplinary cohort study on predictors and consequences of changes in physical, cognitive, emotional, and social functioning in men and women aged 55–85 years at baseline in 1992–93. A random sample stratified for age, sex, and expected 5-year mortality was drawn from the population registries of 11 municipalities in the Netherlands [24]. In 2012–13, the original sample was replenished with 1023 participants aged 55–65 years. The VU University Medical Centre Ethical Review Board approved the study. All participants provided informed consent. As falls data were available in the correct age-range for participants in the 2012–13 cohort only, data from this cohort were used for the current analyses. In total, 862 participants aged 55–64 years with complete data on age, sex, education and falls were included.

The MRC National Survey of Health and Development (NSHD) is an ongoing cohort study of a nationally representative sample of 5,362 males and females born in England, Scotland and Wales during one week in March 1946 [25, 26]. The sample have now been followed up 24 times since birth. Falls data for these analyses were ascertained from nurse interviews during two of the most recent waves of data collection. In 1999, at age 53, 3035 participants were successfully contacted, of whom 2984 received a home visit from a trained nurse. In 2006–2010, at age 60–64, 2856 eligible participants (those known to be alive, living in England, Scotland or Wales and who had not permanently refused to participate) were invited for assessment at one of six clinical research facilities or to be visited by a research nurse at home of whom 2229 were assessed. Relevant ethical approval has been received from the North Thames Multi-Centre Research Ethics Committee (MREC 98/1/121) for the 1999 assessment and from the Central Manchester Local Research Ethics Committee (07/H1008/245) and the Scottish A Research Ethics Committee (08/MRE00/12) for the 2006–2010 assessment. All participants provided informed consent. In total, 2821 participants had complete data on age, sex, educational level attained and falls at age 53 and 2094 had falls data at age 60–64.

The Irish Longitudinal Study on Ageing (TILDA) is an ongoing cohort study designed to achieve a representative sample of community-dwelling people aged 50 years or older in Ireland.[27] A random sample of 25600 residential addresses in Ireland were selected with stratification for socioeconomic status, age and geography. Each address was provided with study information and visited by field staff. All persons aged 50 years and over (primary respondents) and their spouses or partners of any age (secondary respondents) were eligible. Enrolled participants completed a computer-assisted questionnaire, self-completion questionnaire and a health assessment. All participants signed informed consent. Ethical approval has been obtained from the Trinity College Dublin Research Ethics Committee. Baseline data from the 8504 primary and secondary participants were collected between October 2009 and July 2011. For the current analyses, data were used from 4968 participants aged 40–64 years with complete data on age, sex, education and falls.

### Measures

#### Falls

In ALSWH, participants were asked “In the last 12 months, have you: (a) had a fall to the ground?, (b) been injured as a result of a fall?, and (c) needed to seek medical attention for an injury from a fall?”. Participants with a positive response to any one of these three questions were classified as having had a fall. In LASA and TILDA, participants were asked “Have you fallen in the last year?” In the NSHD, participants were asked “Have you fallen at all in the past 12 months?”. Participants who responded ‘yes’ were classified as having had a fall.

#### Socio-economic and health variables

Age, sex, level of education and self-rated health were included to describe the sample. In each cohort, level of education was based on the highest level of qualification attained. The cohort-specific response options were harmonised to match the categories of ‘none or primary education’, ‘secondary education’ and ‘tertiary education’. Although slight variations in wording were used, all cohorts assessed self-rated health with a question similar to “In general, would you say your health is: excellent, very good, good, fair or poor?” The response options were collapsed into ‘excellent-good’ and ‘fair-poor’. The wording of the response options varied slightly in LASA, but were also collapsed into the highest three (excellent-fair) versus the lowest two categories (sometimes good and sometimes bad-poor). BMI was calculated based on height and weight (kg/cm^2^) self-reported in ALSWH and measured in LASA, NSHD and TILDA. Diabetes and arthritis were based on self-report.

### Statistical analyses

The cohorts were described using descriptive statistics. Cohort characteristics were compared using ANOVA for continuous variables and chi-squared test for categorical variables. Crude and weighted prevalence estimates and 95% confidence intervals were calculated for the total sample within each of the cohorts as well as for men and women separately. The weighted prevalence estimates accounted for deviations in the samples from the underlying population at the year of data collection in the distribution of age, sex and level of education. Weights were used to ensure the prevalence estimates were representative of the general population the sample was drawn from. As the Australian cohort included women only, weights were based on age and level of education. In the British cohort, the weights were based on age, sex and fathers occupational status at birth (i.e. manual vs non-manual and agricultural workers) due to lack of availability of comparable census data on education. Finally, prevalence was calculated for each 5-year age-band within each of the cohorts to depict the trend in prevalence with age. Sensitivity analyses were done for 4- and 6-year age-bands to examine the influence of using different age-band widths. Changes in prevalence with age were examined using chi-squared tests. Differences in prevalence by country and sex were examined using chi-squared tests.

## Results

Each cohort has unique features (Table 1). The ALSWH cohort includes a large sample (n = 10556) of women only. The TILDA cohort covers the full age-range of 40–64 years. The LASA cohort includes the most recent data (collected in 2012/13). The ALSWH and NSHD cohorts each have falls data collected at 3 and 2 time points spanning 6 and 10 years of follow-up, respectively.

**Table 1:** Sample characteristics of the four cohorts.

	ALSWH			LASA	NSHD		TILDA	p-value for between-group differences^b^
Country	Australia			Netherlands	Great Britain		Ireland	
N	10556	9547	8992	862	2821	2094	4968	
Survey year	2004	2007	2010	2012–13	1999	2006–10	2010	
Age-range (years)	51–58	53–61	57–64	55–64	53	60–64	40–64	
Age (years, mean (SD))	55.5 (1.5)	58.5 (1.5)	61.5 (1.5)	60.2 (2.8)	53.5 (0.2)	63.3 (1.1)	56.1 (4.8)	<0.001
Sex (% women)	100	100	100	51.6	50.9	52.2	57.5	<0.001
Highest level of education (%)								<0.001
None or primary education	16.5	16.4	15.5	9.7	36.8	31.7	19.6	
Secondary education	48.5	48.3	48.4	58.4	53.3	57.0	45.8	
Tertiary education	35.0	35.3	36.1	31.6	9.9	11.3	34.7	
Self-rated health (%)								0.06
Excellent-Good	85.7	86.3	86.1	87.5	85.4^a^	86.0	85.6	
Fair-Poor	14.3	13.7	13.9	12.5	14.6^a^	14.0	14.4	
BMI (kg/m^2^, mean (SD))	27.2 (5.5)	27.4 (5.6)	27.6 (5.6)	27.1 (4.7)	27.4 (4.8)	28.0 (4.9)	28.6 (5.1)	0.33
Diabetes (%)	4.6	6.7	7.8	7.7	2.9	6.2	5.4	<0.001
Arthritis (%)	26.2	29.5	32.4	40.7	n/a	n/a	19.3	<0.001

ALSWH Australian Longitudinal Study on Women’s Health; LASA Longitudinal Aging; Study Amsterdam; NSHD MRC National Survey of Health and Development; SD standard deviation; BMI body mass index; n/a no comparable data available

^a^ Data were used from the 2006–10 data collection wave.

^b^ Note that the between-group differences were based on data from the 2010 wave in ALSWH and the 2006–2010 wave in NSHD

**Fig 1:**
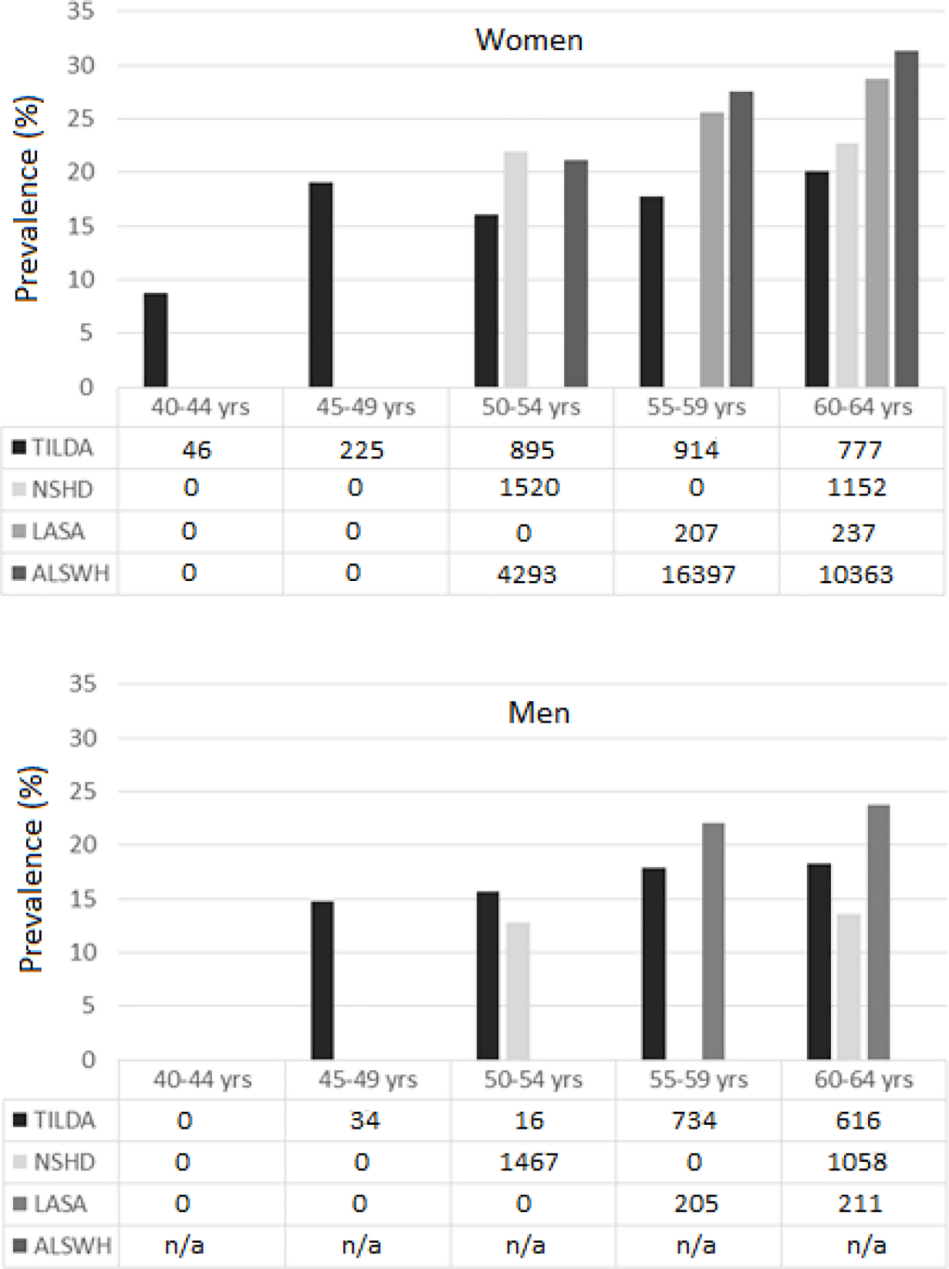
Prevalence of falls per 5-year age band in middle-aged women (top) and men (bottom). Presented are the prevalence of falls per 5-year age bands based on the harmonised data across the four cohorts, and the number of participants providing data in each 5-year age band. Note that ALSWH and NSHD participants could be included more than once if they provided data at multiple data collection waves while still falling within the defined age bands.

Across the cohorts, the crude prevalence of falls in the previous year varied from 12.8% in 53 year old men in Great Britain (NSHD) to 31.4% in 53–61 year old women in Australia (ALSWH) (Table 2). Weighting for age, sex and education had little influence on the prevalence estimates (i.e. less than 2 percentage points). Overall, the prevalence was higher in Australia (27.8%, women only) and the Netherlands (25.1%) than in Ireland (17.6%) and Great Britain (17.8%, p<0.001). Women (27.0%) had higher prevalences than men (15.2%, p<0.001). In women, the prevalence of falls increased with age from 8.7% in 40–44 year olds, 19.1% in 45–49 year olds, 20.9% in 50–54 year olds, 27.3% in 55–59 year olds, to 29.9% in 60–64 year olds (p<0.001). In men, the prevalence of falls increased with age from 14.7% in 45–49 year olds, 13.4% in 50–54 year olds, 19.3% in 55–59 year olds, to 15.7% in 60–64 year olds (p<0.001). However, there is substantial variation in prevalence between the cohorts within any specific age-band (Fig 1). For example, in 60–64 year old women, the prevalence ranged from 20% in TILDA to 31.4% in ALSWH. Sensitivity analyses estimating the prevalence in 4- and 6-year age-bands strengthen the observation that the prevalence of falls increased with age in women, but less so in men (S2 Fig).

**Table 2:** Prevalence of falls in each of the cohorts at each survey.

	ALSWH			LASA	NSHD		TILDA
Country	Australia^a^			Netherlands	Great Britain^a^		Ireland
Year	2004	2007	2010	2012–13	1999	2006–10	2010
N	10556	9547	8992	862	2821	2094	4968
Age-range	51–58	53–61	57–64	55–64	53	60–64	40–64
Crude prevalence of falls (%)							
Total sample	-	-	-	25.1 (22.2–28.0)	17.5 (16.1–18.9)	18.2 (16.6–19.9)	17.6 (16.5–18.7)
Women	22.0 (21.2–22.8)	31.4 (30.5–32.3)	30.4 (29.5–31.4)	27.2 (24.2–30.2)	22.0 (20.5–23.5)	22.8 (21.0–24.6)	17.8 (16.7–18.9)
Men	-	-	-	22.8 (20.0–25.6)	12.8 (11.6–14.0)	13.3 (11.9–14.8)	17.2 (16.2–18.3)
Weighted prevalence of falls (%)							
Total sample	-	-	-	24.4 (21.5–27.3)	17.3 (15.9–18.7)	18.0 (16.4–19.7)	15.9 (14.9–16.9)
Women	21.9 (21.1–22.7)	31.6 (30.7–32.5)	29.4 (28.5–30.3)	27.4 (24.4–30.4)	21.6 (20.1–23.1)	22.7 (20.9–24.5)	16.1 (15.1–17.1)
Men	-	-	-	21.2 (18.5–23.9)	13.4 (12.1–14.7)	14.1 (12.6–15.6)	15.6 (14.6–16.6)

Presented are the crude and weighted prevalence estimates (and 95% confidence intervals). The weighted prevalence accounts for differences between the cohorts and their country-specific population distribution in age, sex and education (where relevant).

^a^ Same sample measured at three (ALSWH) and two (NSHD) time points

## Discussion

Across the four cohorts, the prevalence of falls ranged from 8.7% to 31.1%, and varied by age-group, sex and country. On average, the prevalence was markedly higher than the 11.4% found in 45–64 year old adults in the 2008 U.S. National Health Interview Survey[4] and the 2-year prevalence of 21% in 45–64 your old adults in the Baltimore Longitudinal Study of Aging [14]. In addition to age and sex, factors that explain this variation in prevalence between countries include variations in prevalence of key fall risk factors and the measurement of falls. This will be discussed in the following paragraphs. Regardless of the differences in absolute prevalences between the countries, there is consistent evidence of an increase in prevalence of falls across mid-life, particularly in women.

Age is the factor most likely to explain the variation in prevalence between the countries. As shown in the current and previous studies, falls risk increases with age [1, 28]. One would therefore expect the prevalence to be higher in cohorts with older average age. Indeed, the prevalence was higher in cohorts that were older at the time of assessment (Table 2). However, even within 5-year age-bands, substantial variation in prevalence was found between the cohorts. For example, within the 60–64 year old women, the prevalences were 20.1% in Ireland, 22.7% in Great Britain, 28.7% in The Netherlands and 31.1% in Australia (Fig 1). This suggests that other factors also play a role, such as socio-demographic, health and lifestyle factors.

In adults over the age of 65, women typically have a higher prevalence of falls than men [2, 28]. For example, a meta-analyses of observational studies showed that women have a 1.3 times higher odds of falls than men [28]. The current study also confirms this for 40–64 year olds. The sex-differences in prevalence were more pronounced in the Netherlands and Great Britain than in Ireland (Table 2). The Australian cohort included women only and also reported the highest prevalence. It therefore seems likely that differences in sex distribution contribute to the variation in prevalence between countries. A potential explanation for the stronger increase in prevalence of falls during midlife in women than men may be explained by the concurrent stronger increase in prevalence of risk factors for falls, such as arthritis and cardiovascular diseases, post menopause in women.

The four cohorts were designed to be nationally representative and included mainly Western-Europeans. The Australian cohort included mostly women born in Australia of British or Irish descendency (77.8%). Few studies have examined ethnic differences in falls risk of older adults and the findings are inconsistent, but there is some evidence that falls risk may be lower in African Americans than in White Americans [16, 18] and lower in Asians than in Caucasians globally [16, 17]. Hence the current results may not be generalizable to other populations with different ethnic backgrounds.

Many risk factors for falls have been identified in older adults. It is yet unclear whether the same risk factors are important in middle-aged adults. In older adults, some of the strongest risk factors include mobility limitations and chronic conditions such as diabetes and arthritis [28]. Indeed, the prevalence of diabetes and arthritis were higher in the Dutch cohort than in the British and Irish cohorts (Table 1) and could partly explain the higher prevalence of falls in this cohort. In contrast, the prevalence of arthritis was lower in the Australian cohort than in the Dutch cohort, while the prevalence of falls was higher in the Australian cohort. A previous study by our group found that, diabetes was not identified as a risk factor of falls in Australian middle-aged women, but presence of joint symptoms was [15]. That study also found that different factors were associated with falls at early, middle and later midlife, highlighting the complexity of falls risk at this life stage [15]. For example, high levels of alcohol intake and hearing problems were significant predictors of falls at ages 59–67, but not at earlier ages [15]. The differences found by age, sex and country could be explained by variations by age, sex and country in risk factors for falls. Further research is required to identify key risk factors of falls in middle-age.

Finally, the variation in prevalence between the cohorts may also be partially explained by differences in measurement of falls. While there was little variation in the wording used to ask participants about falls in the past year, there were variations in how the questions were presented. The Irish, British and Dutch cohorts use a conditional approach whereby follow-up questions about the consequences of a fall are only asked if the participant responds positively to the first question about a fall in the past year. In the Australian cohort, the question specifically asked about ‘falls to the ground’ and the follow-up questions about the consequences of a fall are visible in the survey to all participants. It may be that the specific wording and these follow-up questions facilitate the recall of falls. Moreover, in the Australian cohort, some participants responded ‘yes’ to either ‘having had an injury from a fall’ or ‘seeking medical attention after a fall’, but not to the actual falls question. As one cannot have an injury from a fall without actually having a fall, these participants were classified as fallers. This resulted in a 2–2.6 percentage point increase in the prevalence of fallers at each survey. Follow-up questions about the consequences of falls may help improve recall and reduce misclassification.

Falls are currently perceived as a major problem in older adults, but receive little attention in middle-age. While the prevalence of falls is *lower* in middle-aged adults than in older adults, the current findings show that the prevalence is not *low*. Previous studies have suggested a U-shaped association between age and fall risk, in which the fall risk is highest in children and seniors [29]. This may result in the misperception that falls are not of concern in middle-aged adults. While falls that result in injuries have the greatest impact on health services use, any fall, with or without injury, can result in fear of falling and avoidance of physical or social activities [20, 30]. Therefore all falls have the potential to influence wellbeing [20]. Repeating the analyses for ‘falls requiring medical attention’ (S2 Fig) showed similar trends as for all falls (Fig 1). However, data were available only for the Irish and Australian cohorts, and for the 2006–2010 assessment in the British cohort. On average, a third of all reported falls (32%) required medical attention,which is similar to the proportion reported for older adults [14, 30]. Moreover, the current data show a marked increase in prevalence from 8.7% at age 40–45 to 19.1% at age 45–50 in Irish women (Fig 1). The timing of this increase in falls coincides with the onset of menopause [31], decline in balance performance [11] and increase in prevalence of syncope and vertigo [12, 13]. These collective findings warrant further research to inform preventive strategies in middle-aged adults. A better understanding of the factors that drive this increase in fall risk in middle-age may be the key to effective preventive interventions earlier in life with potential sustained benefits into older age.

### Strengths and limitations

Strengths of this study include the use of data from four population-based cohort studies with a total of 19207 participants. The large sample enabled subgroup comparisons by age and sex and provided insight into variations in prevalence estimates across countries. As is common in cohort studies, participants included in the analyses are likely healthier than those who refused to participate or dropped out [32]. The current prevalence estimates may therefore underestimate the true prevalence of falls in 40–64 year old adults. By weighting the prevalence estimates for age, sex and education, we compensated for potential selective drop-out or deviations in the cohorts from the national distribution. The weighted prevalence estimates were slightly lower (<2 percentage points) than the crude prevalence rates. This is likely to be explained by the underrepresentation of participants at the lower end of the age-range. Another limitation of this study is that the falls data were based on self-report. All cohorts used a 12-month recall, which has 89% agreement with medical records data [33]. Although calendar-based methods are preferred, underreporting of falls is an issue with all methods used [34].

### Conclusions

Across the four cohorts, the annual prevalence of falls ranged from 8.7% to 31.1% depending on age-group, sex and country. The prevalence was higher in women than in men and increased with age. There was substantial variation in the prevalence of falls between the four countries, even within 5-year age-bands, which are likely explained by differences in sample characteristics such as age, sex, ethnicity and chronic conditions. The sharp increase in prevalence of falls in middle-age, particularly among women, supports the notion that falls are not just a problem of old age, and that middle-age may be a critical life stage for preventive interventions. Further research to inform preventive strategies in middle-aged adults is warranted.

## Supporting information

S1 Fig **Prevalence of falls per 4- and 6-year age bands in middle-aged women (top) and men (bottom).** Presented are the prevalence of falls per 4-year age bands (left panels) and 6-year age bands (right panels) based on the harmonised data across the four cohorts, and the number of participants providing data in each age band. ALSWH participants could be included more than once if they provided data at multiple data collection waves while still falling within the defined age bands.
(TIF)

S2 Fig **Prevalence of falls requiring medical attention per 5-year age band in middle-aged women (top) and men (bottom).** Presented are the prevalence of falls requiring medical attention per 5-year age bands based on the harmonised data across the four cohorts, and the number of participants providing data in each 5-year age band. Note that data were available for ALSWH, NSHD (wave 2006–2010 only) and TILDA, but not for LASA. ALSWH participants could be included more than once if they provided data at multiple data collection waves while still falling within the defined age bands.
(TIF)
