# Supplementary figures and images for: Should prevention of falls start earlier? Co-ordinated analyses of harmonised data on falls in middle-aged adults across four population-based cohort studies
Source: PLoS One. 2018 Aug 7;13(8):e0201989. doi: 10.1371/journal.pone.0201989 (PMC6080796; doi:10.1371/journal.pone.0201989)

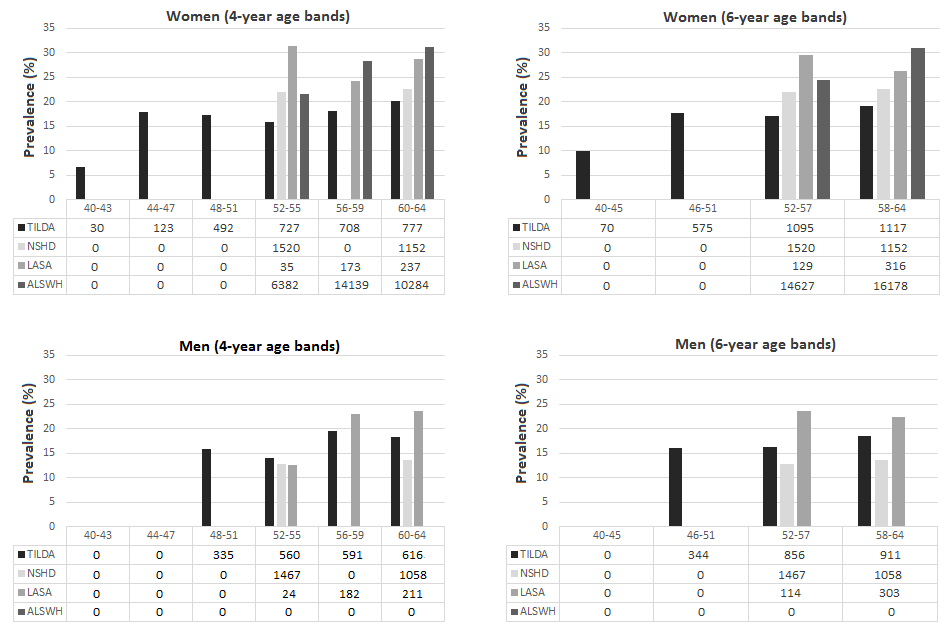

Supplement: S1 Fig — Prevalence of falls per 4- and 6-year age bands in middle-aged women (top) and men (bottom). Presented are the prevalence of falls per 4-year age bands (left panels) and 6-year age bands (right panels) based on the harmonised data across the four cohorts, and the number of participants providing data in each age band. ALSWH participants could be included more than once if they provided data at multiple data collection waves while still falling within the defined age bands. (TIF) [file pone.0201989.s001.tif]

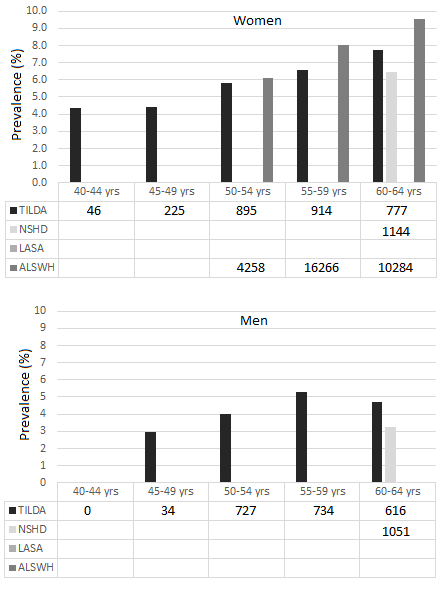

Supplement: S2 Fig — Prevalence of falls requiring medical attention per 5-year age band in middle-aged women (top) and men (bottom). Presented are the prevalence of falls requiring medical attention per 5-year age bands based on the harmonised data across the four cohorts, and the number of participants providing data in each 5-year age band. Note that data were available for ALSWH, NSHD (wave 2006–2010 only) and TILDA, but not for LASA. ALSWH participants could be included more than once if they provided data at multiple data collection waves while still falling within the defined age bands. (TIF) [file pone.0201989.s002.tif]
